# Bridging the Gap: A Secondary Data Analysis of Implementation Outcomes and Symptom Trajectories of an eHealth Mental Health and Parenting Treatment Among Mothers With and Without At-Risk Substance Use

**DOI:** 10.1177/29768357261438586

**Published:** 2026-04-09

**Authors:** Kayla M. Joyce, Robert J. W. McHardy, Lauren E. Kelly, Kristin Reynolds, Natalie Mota, Lianne M. Tomfohr-Madsen, Leslie E. Roos

**Affiliations:** 1 Department of Clinical Health Psychology, University of Manitoba, Winnipeg, MB, Canada; 2 Department of Psychology, University of Manitoba, Winnipeg, MB, Canada; 3 Department of Pharmacology and Therapeutics, University of Manitoba, Winnipeg, MB, Canada; 4 Department of Educational and Counselling Psychology, University of British Columbia, Vancouver, BC, Canada; 5 Department of Pediatrics, University of Manitoba, Winnipeg, MB, Canada; 6 Children’s Hospital Research Institute of Manitoba, Winnipeg, MB, Canada

**Keywords:** eHealth, mothers, postpartum, mental health, substance use, cannabis, alcohol

## Abstract

**Background:**

Several eHealth treatments, including the Building Emotional Awareness and Mental Health (BEAM) program, are feasible and effective for mothers with mental health concerns such as depression and/or anxiety. BEAM is a mental health and parenting program that does not directly address substance use. Prior BEAM trials observed high rates of alcohol and cannabis use among participants, despite not recruiting based on substance use. No research has examined BEAM among mothers with mental health concerns and at-risk substance use (ARSU), highlighting the need to understand implementation metrics and symptom trajectories among mothers with depression/anxiety and ARSU (ARSU+) and without ARSU (ARSU−).

**Objectives:**

To address this gap, we examined BEAM implementation metrics (retention, session attendance, perceived usefulness, safety) and mental health and parenting stress symptom trajectories over time in mothers with ARSU+ and ARSU−.

**Design:**

This was a secondary data analysis of 2 BEAM trials for mothers of infants (6-18 months) and toddlers (18-36 months).

**Methods:**

One-hundred-and-ten participants (*M*_age_ = 31.85) from the BEAM arm of 2 trials: infant (n = 40) and toddler trials (n = 70). Implementation metrics were evaluated against pre-specified benchmarks. Mental health and parenting stress symptom trajectories were examined across time using 2-level mixed models.

**Results:**

Treatment adherence and safety benchmarks were met in both groups, the perceived usefulness benchmark was met only in the ARSU+ group, and the retention benchmark was not met for either group. Mental health and parenting stress symptom trajectories were largely similar between ARSU subgroups over time.

**Conclusion:**

This study extends evidence on BEAM by showing that mothers with ARSU+ can safely engage with BEAM, have acceptable treatment adherence, and describe BEAM as useful. Retention remains the key implementation barrier for mothers with ARSU+. Future BEAM iterations should prioritize retention-focused supports (eg, proactive engagement, incentives) to strengthen real-world impact.

## Introduction

Depression/anxiety and at-risk substance use (ARSU), defined here as hazardous patterns of alcohol and/or cannabis use identified by established screening cutoffs,^1,2^ are common and often co-occur during the postpartum period, posing risks for mothers and their infants. (Please note: In this study, the term ARSU is used to refer to mothers who met established screening cutoffs for hazardous alcohol and/or cannabis use on the Alcohol Use Disorder Identification Test (AUDIT) and/or Cannabis Use Disorder Identification Test – Revised (CUDIT-R).^1,2^ Mothers who did not meet these cutoffs are referred to as ARSU−. ARSU reflects hazardous use defined on a screening measure and does not necessarily indicate a diagnosed substance use disorder. We use the term “at-risk” to denote elevated risk of substance use-related harms consistent with AUDIT/CUDIT-R interpretation, not to imply a global deficit or risk across domains.) An estimated 10% to 20% of mothers experience postpartum depression,^3^ 14.9% engage in binge drinking,^4^ and 4.1% use cannabis while lactating.^5^ Among those in substance use treatment, nearly 70% report a history of depression,^6^ and nearly 1-in-5 postpartum mothers with ARSU experience co-occurring depressive and/or anxiety symptoms.^7^ Given approximately 80% of women with substance use disorders who were abstinent in the last month of their pregnancy relapsed to at least one substance postpartum (ie, tobacco, alcohol, cannabis, or cocaine),^8,9^ the postpartum period represents a high-risk window for ARSU relapse. However, mothers with co-occurring depression/anxiety and ARSU (ARSU+) are often underrepresented in mental health treatment research.^10^ Restrictive clinical trial exclusion criteria, reliance on treatment-seeking samples, and stigma-related barriers to disclosure of substance use likely contribute to the underrepresentation of mothers with ARSU+ in mental health research settings.^11-13^ Consequently, it remains unclear whether scalable, evidence-based mental health and parenting programs are feasible and acceptable for addressing maternal mental health symptoms and parenting stress, given much of the literature is based on treatment-seeking samples with diagnosed substance use disorders and may not generalize to this group.^14^ This underscores the need to examine whether these treatments are feasible and have potential to impact mental health and parenting stress symptom trajectories for mothers with ARSU+.

### The Case for Scalable and Feasible Mental Health and Parenting Programs

The need for scalable, accessible mental health and parenting programs is particularly pressing for mothers with ARSU+. Rates of maternal ARSU+ increased during the COVID-19 pandemic, with evidence of sustained elevations, yet few studies have examined how evidence-based mental health and parenting programs perform for this group, and even fewer have evaluated their feasibility in real-world settings.^15-17^ This gap represents a missed opportunity to examine both the feasibility of these scalable programs and trajectories of mental health symptoms and parenting stress among mothers with ARSU+.

High-intensity integrated perinatal substance use disorder treatments, combining addiction treatment with on-site pregnancy-, parenting- and child-focused services (eg, maternal mental health services, parenting education, childcare, wraparound services), are available, particularly in residential settings.^18^ Reviews suggest these integrated approaches yield small benefits over non-integrated approaches for parenting^19^ and substance use outcomes,^20^ but they are typically resource intensive and setting-bound. As a result, they may have limited reach for mothers with ARSU+ who may benefit from integrated mental health and parenting treatments delivered in more accessible, lower-intensity formats.^21^ This highlights the value of stepped-care pathways that can tailor treatment intensity to need, including low-intensity integrated mental health and parenting skill options when appropriate.^21^

Across treatment intensity levels, engagement can be undermined by stigma and fear of child welfare involvement, alongside practical constraints such as transportation, scheduling, and childcare.^22^ These barriers are concerning given links between emotional distress, parenting stress, and substance use, often via emotion dysregulation and coping-motivated substance use as described in self-medication theory.^23-26^ Consistent with this, co-occurring substance use may be associated with attenuated treatment response and reduced gains, particularly when barriers interfere with sustained engagement and follow-up.^11,22,27^ As a first step toward identifying scalable treatments for mothers with ARSU+, it is important to evaluate implementation outcomes (ie, retention rate, perceived usefulness, treatment adherence, safety) and examine whether evidence-based programs are feasible and associated with improved mental health and parenting stress symptom trajectories in this group.

### Emerging Evidence for eHealth Treatments

Although mental health and parenting programs show promise, they remain limited in availability and are often resource-intensive, requiring specialized staff and in-person delivery formats that may be inaccessible for many families.^19,28^ These limitations have prompted growing interest in eHealth programs which may reduce common barriers including childcare, geographic location, and long waitlists.^29^ Acceptable levels of engagement, retention, and satisfaction have been reported in eHealth programs for parents of children aged 0 to 5 years.^30^ Meta-analyses of eHealth programs show small-to-medium effects for reducing parental depression, anxiety, and stress,^30^ as well as small effects for reducing depression and anxiety symptoms during pregnancy.^31^ These findings suggest eHealth programs are feasible with potential for scalable delivery to mothers of young children. One promising eHealth program that incorporates mental health and parenting skills is the Building Emotional Awareness and Mental Health (BEAM) program.

### The BEAM Program

BEAM is an eHealth program designed to support mothers by integrating mental health and parenting skills. Co-designed with a parent advisory board (PAB) of mothers with lived experience of postpartum depression/anxiety,^32^ BEAM connects therapists and mothers in a secure, accessible online setting. BEAM merges telehealth best practices with peer connection to address key treatment barriers and is preferred by parents.^33-35^ Core components of BEAM include: (a) 2 weekly expert-led psychoeducational videos (mental health and parenting skills), adapted from the Unified Protocol and emotion-focused parenting strategies^36-38^; (b) weekly telehealth groups to consolidate content, discuss challenges and successes, and build a sense of community^39,40^; (c) an anonymous, closed community forum to reflect on skills, share experiences, and promote peer connection^34,41,42^; (d) symptom monitoring to enhance emotional understanding^43-47^; and (e) suggested assignments to support real-world skill application.^48^ BEAM engagement has been high across trials, with most mothers reporting satisfaction with its content, usability, and overall usefulness.^33,35,49,50^ Sociodemographic predictors of increased engagement include older maternal age and higher household income, while being a first-time parent is associated with lower engagement.^51^

BEAM’s effectiveness has been evaluated in 4 trials involving mothers of children aged 6 to 36 months. None of the trials excluded mothers with ARSU+, but ARSU was not systematically assessed as an outcome or subgroup. Two studies targeted mothers of children aged 6 to 18 months,^33,49^ and two focused on mothers of children aged 18 to 36 months.^35,50^ For mothers of infants aged 6 to 18 months, an open-pilot trial (n = 46) reported medium-to-large improvements in maternal depression (*d* = 0.93), anxiety (*d* = 0.58), and parenting stress (*d* = 0.63).^33^ A subsequent feasibility randomized controlled trial (RCT; n = 80) compared BEAM to MoodMission, another evidence-based eHealth program.^52-57^ Both groups showed improvements in mental health symptoms (depression, anxiety, sleep disturbance, anger) and parenting stress, but BEAM participants experienced greater reductions in anxiety after controlling for enrollment symptom severity.^49^ Among mothers of toddlers aged 18 to 36 months, a pilot non-superiority trial found feasibility, with moderate reductions in maternal depression (*d* = 0.47) and parenting stress (*d* = 0.33); 66.7% of participants showed clinical improvements in depression and 52.9% in anxiety.^50^ Finally, a large RCT (n = 119) also found greater reductions in anxiety for mothers in BEAM versus those in treatment as usual (TAU), although depressive symptoms improved similarly across both groups.^50^

BEAM’s transdiagnostic design, eHealth format, focus on mental health and parenting stress, emphasis on peer connection, and inclusion of an anonymous community forum may make it a promising approach for mothers with ARSU+. These features may help reduce stigma and logistical barriers, while supporting emotional regulation and parenting skills. However, BEAM outcomes have not been evaluated by ARSU subgroup, and its feasibility and symptom trajectories among mothers with ARSU+ remain unclear. Accordingly, the primary aim of this secondary data analysis was to assess implementation outcomes for mothers with ARSU+ and ARSU− within BEAM. Exploratory analyses also examined mental health and parenting stress symptom trajectories to characterize change over time among BEAM participants, rather than evaluating BEAM’s efficacy versus TAU.

### The Current Study

Pre-intervention data from Roos et al^50^ indicated that even among BEAM participants not recruited for ARSU+, 1 in 7 met screening cutoffs for ARSU (score ≥8 on the AUDIT^1^), consistent with hazardous alcohol consumption, and 1 in 3 reported using cannabis on 25+ days in the past month (*M* = 4.9 standard joints/day).^1,50,58^ Findings underscore the need to examine whether BEAM is feasible for mothers with ARSU+ and whether mental health and parenting stress symptom trajectories differ between ARSU subgroups. We therefore conducted a secondary analysis of participants in the BEAM arm pooled from 2 RCTs (infant trial: children aged 6-18 months; toddler trial: children aged 18-36 months)^49,50^ to examine implementation outcomes by ARSU subgroup and assess mental health and parenting stress symptom trajectories within BEAM. Since this analysis was restricted to participants in the BEAM arm and was not powered for between-condition comparisons, we focused on within-BEAM implementation outcomes and mental health and parenting stress symptom trajectories by ARSU subgroup. (Although this power analysis was conducted after data collection, it differs fundamentally from traditional post hoc power calculations, which have been widely criticized for relying on observed p-values and/or effect sizes, and for offering no new inferential information.^59^ Instead, this power analysis used a simulation-based approach via the simr package in R,^60^ which prospectively estimates the ability to detect a theoretical time*ARSU group interaction effect (β = .30) given the study design (ie, sample size, timepoints, missingness, variance structure). In other words, given the structure of the collected data, could the study have detected a meaningful effect if one were present? Although this analytic model initially included a random slope for time, the simulation was based on a simplified random intercept only model due to convergence issues and singular fit warnings when including random slopes. Under this simplified model, the estimated power to detect a moderate time*ARSU group interaction was only 4% (95% CI = 1.1%-9.9%), demonstrating that the available sample size was insufficient to detect between-condition differences. Based on this evidence, comparisons between BEAM and TAU ARSU+ participants were not discussed in this paper.)

Based on prior perinatal and substance use research indicating that substance use is associated with lower engagement and higher attrition due to barriers such as stigma, fear of child welfare involvement, unstable housing, and competing caregiving demands,^11,22,27^ we anticipated that mothers with ARSU+ would show less favorable implementation outcomes compared to those with ARSU−.^11,22^ Both ARSU subgroups were also expected to show reductions in mental health symptoms and parenting stress over time, consistent with prior BEAM findings,^33,35,49,50^ but we also explored whether symptom improvements over time differed by ARSU subgroup, given evidence linking co-occurring substance use with attenuated gains in mental health and parenting programs.^11,22,27^

## Methods

### Trial Design

Participants (N = 110) were BEAM-arm mothers pooled from 2 RCTs: the *infant trial*,^49^ which compared 10 weeks of BEAM (mental health and parenting treatment; n = 40) with MoodMission (mental health-only treatment)^52-57^; and the *toddler trial*,^50^ which compared 10 weeks of BEAM (n = 70) to TAU (a list of community mental health resources and no restrictions on service use). BEAM used the same treatment, core procedures, outcome measures, and follow-up periods in both trials, supporting their integration for the purposes of this secondary data analysis. Mothers were recruited between January and March 2022 for the infant trial, and between November 2021 and February 2022 for the toddler trial. Participants were recruited using paid, targeted advertisements on social media platforms. For the infant trial, recruitment also occurred via poster distribution through the listserv of a local community agency and in locations frequented by mothers (eg, libraries). Participants were randomized at a 1:1 ratio using computer-generated randomization and were allocated by an independent research assistant.^61,62^

Both RCTs were registered with ClinicalTrials.gov (NCT05398107 and NCT05306626) and the protocols are published.^61,62^ Ethics approval was obtained from the University of Manitoba’s Research Ethics Board (REB) for both trials (REB#: HE2021-0217 and P2020:081) and were conducted according to the Declaration of Helsinki.

### Participants

To be eligible for the infant trial, participants had to: (a) be a woman-identifying primary caregiver (biological, adoptive, foster, stepparent) (Participants had to identify as a mother regardless of their sex assigned at birth); (b) be ≥18 years old; (c) have an infant 6-18 months old at recruitment; (d) reside in Manitoba, Canada; (e) obtain a score ≥10 on the Patient Health Questionnaire – 9 (PHQ-9)^63^ or Generalized Anxiety Disorder – 7 (GAD-7)^64^; (f) complete the Mini International Neuropsychiatric Interview (MINI)^65^ and meet diagnostic criteria for a major depressive episode and/or anxiety disorder; (g) complete pre-intervention (T1) questionnaires; (h) be available to attend weekly telehealth groups; and (i) comfortably communicate in English (spoken and written). Since BEAM’s effectiveness has not been assessed for certain mental health concerns, participants were excluded if they: (a) self-reported a suicide attempt (past year) or self-harm behaviors (past 6 months) or (b) screened positive for psychotic symptoms, post-traumatic stress disorder, or an alcohol/substance use disorder on the MINI.^65^ A MINI diagnosis alone was not an automatic exclusion, and participants were excluded only when, based on clinical judgment, symptom severity were expected to interfere with safe participation in BEAM or indicated a need for higher-level treatment. (No participants were excluded due to endorsement of psychotic symptoms, post-traumatic stress disorder, or an alcohol/substance use disorder on the MINI^65^). Excluded participants were provided with resources and referred to services better suited to their needs.

Eligibility criteria for the toddler trial were similar to the infant trial except participants: (a) had a toddler 18-36 months old at recruitment; (b) resided in Manitoba or Alberta, Canada; (c) consented to wearing a Fitbit (unrelated to this study); and (d) completed a Zoom assessment and/or orientation session.

### The BEAM Program

BEAM is a 10-week guided mental health and parenting skills program where mothers progress through weekly modules alongside a cohort of mothers with depression and/or anxiety. BEAM teaches skills designed to increase supportive parenting behaviors and promote maternal-child bonding, with the goal of disrupting theorized mechanisms of environmental risk transmission.^32,66^ It includes 5 weekly components (see introduction). BEAM was supported by clinical, parent, and peer coaches. Clinical coaches were graduate-level clinical psychology students who were supervised by a clinical psychologist, led/co-led telehealth groups, and managed participant communication. Peer coaches were previous participants with lived experience who supported participants^32^ and were compensated for their time. Parent coaches (infant trial only) were trained mental health professionals from a local community agency. They co-led telehealth groups and engaged on the community forum.

### Measures

#### Participant Characteristics

##### Sociodemographics

Maternal, child, and household demographics were collected to characterize the sample. Breast/chest feeding status was assessed using an author-compiled item administered at T1, post-intervention (T2), and 6-month follow-up (T3).

##### Substance Use Questionnaire

Past 6 month substance use frequency was assessed using the AUDIT^1^ and CUDIT-R.^2^ Cannabis use quantity was assessed using a standardized joint.^58^ Information on tobacco and illicit drug use was also collected.

#### Implementation Measures

Implementation metrics were derived from the Research Electronic Data Capture (REDCap) platform.^67,68^ Data was assessed descriptively between ARSU subgroups and included: retention rate, perceived usefulness, treatment adherence, and safety (Table 1 includes definitions and benchmarks). Each outcome was evaluated against a pre-specified benchmark.^61^

**Table 1.** Implementation Metrics.

Implementation metric	Indictor	Benchmark	ARSU+	ARSU−
			M(SD)/%	M(SD)/%
Retention rate	% of mothers with data at follow-up	⩾80% of mothers with follow-up data	69.2%	77.4%
Perceived usefulness	Usefulness subscale of mHealth App Usability Questionnaire^a^	Mean score of ⩽3	**2.6 (1.8)**	3.2 (1.8)
Treatment adherence	Logging onto the BEAM App and/or attending weekly group telehealth session	Participation in >50% of program weeks via logging onto the BEAM App or attending weekly telehealth group sessions	**84.6**%	**89.0**%
Safety	Adverse events from the BEAM program or assessments	No adverse events reported	**0 adverse events reported**	**0 adverse events reported**

*Note.* Results meeting pre-specified criteria for success are in bold.

a Zhou et al.^69^

##### mHealth App Usability Questionnaire (MAUQ)

The MAUQ assessed perceptions of BEAM^69^ and included 3 subscales, which demonstrated adequate internal consistency: ease of use (α = .94), interference/satisfaction (α = .94), and perceived usefulness (α = .90).

#### Clinical Outcome Measures

##### AUDIT

The AUDIT is a valid and reliable measure used to assess alcohol consumption, drinking behaviors, and alcohol-related problems (α’s = .69-.78).^1,70^ Consistent with international guidelines, total scores of 8 to 15 indicated hazardous alcohol use and >15 indicated the likelihood of alcohol dependence.^1^ A cutoff of ≥8 identified mothers whose alcohol use was at least hazardous, reflecting a drinking pattern associated with an increased risk of health and social harms (eg, binge drinking, impaired functioning).^1^

##### CUDIT-R

The CUDIT-R is a valid measure of cannabis use (α’s = .79-.85),^2^ with total scores between 8 and 11 indicating hazardous cannabis use and ≥12 suggesting a probable Cannabis Use Disorder.^2^ A cutoff of ≥8 identified mothers whose cannabis use was at least hazardous, highlighting use characterized by frequency and cannabis-related problems (eg, difficulty cutting down, using despite negative consequences).^2^

##### GAD-7

Anxiety symptom severity over the past 2 weeks was assessed using the GAD-7.^64^ Total scores ⩾10 represented moderate to severe anxiety.^64^ The GAD-7 demonstrated adequate internal consistency (α’s = .88-.90).

##### MINI

The MINI is a structured diagnostic interview which assessed the presence of mental health disorders (infant trial only).^65^ Pre-specified modules were administered including major depressive episode, suicidality, generalized anxiety disorder, panic disorder, agoraphobia, social anxiety disorder, post-traumatic stress disorder, alcohol use disorder, substance use disorder, and psychotic disorder and mood disorders with psychotic features. The MINI is based on the Diagnostic and Statistical Manual of Mental Disorders (DSM), Fifth Edition^71^ which was adapted from the DSM-IV.^72^ Psychometric properties of the MINI DSM-5 version are not published; however, good interrater and test-retest reliability is shown for the MINI DSM-IV version.^65,73^

##### PHQ-9

Depressive symptom severity over the past 2 weeks was assessed using the PHQ-9.^63^ Total scores ⩾10 represented moderate to severe depression.^63^ The PHQ-9 demonstrated adequate internal consistency (α’s = .78-.88).

##### Parenting Stress Index – Short Form (PSI-SF)

The Parent-Child Dysfunctional Interaction subscale of the PSI-SF examined the extent parents felt satisfied with their child and interactions with their child.^74^ Higher total scores indicated greater dysfunction in the parent-child relationship. This subscale had adequate internal consistency (α’s = .80-.83).

##### Patient-Reported Outcomes Measure Information System (PROMIS) – Anger

The PROMIS – Anger assessed anger, negative social cognitions, and efforts to control anger.^75^ Higher total scores indicated greater anger-related distress. The PROMIS – Anger demonstrated adequate internal consistency (α’s = .82-.91).

##### PROMIS – Sleep Disturbance

The PROMIS – Sleep Disturbance examined participants’ perceptions on their sleep quality, depth, and restorative value.^76^ Higher total scores indicated greater sleep disturbances. The PROMIS – Sleep Disturbance demonstrated adequate reliability (α’s = .89-.90) and is valid.^77,78^

### Procedure

Table 2 depicts the schedule of enrollment, interventions, and assessments for both trials.^79,80^ At enrollment (T0), mothers completed an online screening questionnaire that included sociodemographic information, a suicidality/self-harm screener, PHQ-9,^63^ and GAD-7^64^ to determine initial eligibility. Informed consent was obtained electronically via the REDCap^67,68^ platform prior to completing the T0 eligibility screener, before the MINI (infant trial only), and before study participation. Eligible participants were invited to complete self-report measures via REDCap^67,68^ at T1, T2, and T3. Compensation ≤$160 CAD was provided in both trials, although compensation structure differed slightly. In the infant trial, participants were compensated: $25 for the MINI, $25 for T1 questionnaires, $50 for T2 questionnaires, $25 for T3 questionnaires, $25 for completing >75% of the weekly surveys, and $10 for those who completed the T2 and T3 questionnaires within 1 week of distribution. In the toddler trial, participants were compensated: $30 for T1 questionnaires and the Zoom assessment, $30 for T2 questionnaires, $30 for T3 questionnaires, and ≤$30 for weekly surveys ($3 per survey or $30 for all weeks). Participant were offered $50 for returning their Fitbit; alternatively, they could opt to keep the Fitbit in lieu of this $50 compensation.

**Table 2.** Standard Protocol Items: Recommendations for Interventional Trials (SPIRIT) Schedule of Enrollment, Interventions, and Assessments.

	Enrollment (T0)	Pre-Intervention (T1)	Allocation	Intervention	Post-intervention (T2)	Follow-up (T3)
Study procedures	Week 1	Weeks 1-9	Week 9	Weeks 9-19	Weeks 19-21	Weeks 32-34
Informed consent	X					
Eligibility screener	X					
Psychodiagnsotic mental health assessment^a^	X					
Allocation to treatment or control			X			
BEAM program				X		
Treatment outcome questionnaires		X			X	X

a The psychodiagnostics interview, that is, the Mini International Neuropsychiatric Intervention,^65^ was only conducted with mothers in the infant trial.

### Data Analysis

#### Sample Size

A sample size calculation was not conducted, as this secondary analysis was exploratory and implementation-focused, and subgroup power calculations are not appropriate in this context.^81^

#### Scoring and Variable Preparation

Prior to conducting analyses, participants were subdivided into ARSU group based on their AUDIT and CUDIT-R scores. Participants were categorized into the ARSU+ subgroup if they scored ≥8 on the AUDIT and/or CUDIT-R at T1.^1,2^ This threshold corresponds with established cutoffs for hazardous alcohol and cannabis use on the AUDIT and CUDIT-R.^1,2^ Participants who did not meet either cutoff at T1 were categorized as ARSU−. For each mental health and parenting stress measure, percent possible scores were calculated by subtracting the scale’s minimum possible score from each participant’s score and then dividing the result by the scale’s maximum possible score. A composite mental health score was then calculated by averaging the percent possible scores across all 4 mental health measures (PHQ-9, GAD-7, PROMIS – Anger, PROMIS – Sleep Disturbance).^50^

#### Implementation Metrics

All implementation metrics were descriptively compared between ARSU subgroups to the a pre-specified implementation metric benchmark (Table 1 includes definitions and benchmarks).^61^ Post-hoc descriptive analyses were conducted on self-reported BEAM technological difficulties (infant trial only) to provide possible result explanations.

#### Symptom Trajectories

Descriptive and inferential statistics were conducted in the Statistical Package for the Social Sciences (SPSS; version 29).^82^ α = .05 indicated statistical significance. Given the study’s exploratory nature, *P*-values were not adjusted for multiple comparisons.^83,84^ Independent sample *t*-tests and chi-square tests were used to examine ARSU subgroup differences at T1. Bivariate correlations were conducted between T1 symptom levels, symptom change scores (T2-T1, T3-T1) and sociodemographic variables that differed between ARSU subgroups at T1.

All participants randomized to the BEAM arm were included in analyses regardless of adherence. Two-level linear mixed models with random intercepts and slopes were conducted in MPlus using the maximum likelihood estimator robust to non-normality (version 8.4)^85^ to examine the effect of group (ARSU+ = 1, ARSU− = 0) on symptom change across time (T1-T3). Models examined main effects of ARSU subgroup and time (T1-T3) on mental health and parenting stress symptoms, and ARSU subgroup as a moderator of the symptom change slope across time (T1-T3). Sociodemographic factors that differed by ARSU subgroup at T1 were included as model covariates (see Supplemental Materials). Using Glass’ Δ,^86^ effect sizes for ARSU subgroup on slope of symptom change are presented to contextualize between-group differences in symptom change, and are interpreted as 0.20 (small), 0.50 (medium), and 0.80 (large).^87,88^

A second set of models added T0 symptom severity to the first models. T0 symptom severity was calculated as the average percent possible score for the PHQ-9 and GAD-7 at T0 (ie, an indicator of internalizing symptoms). T0 symptom severity was added as a control (direct effect) of symptoms across time (T1-T3). Symptom severity was also added as a moderator of symptom level (T0 symptom severity*ARSU group) and of the slope of symptom change across time (T0 symptom severity*time), which allowed for an examination of whether T0 symptom severity was differentially associated with symptoms by ARSU subgroup and whether symptom change differed by T0 symptom severity. Finally, T0 symptom severity was added in a 3-way T0 symptom severity*ARSU subgroup*time interaction to examine whether T0 symptom severity differentially affected the slope of symptom change between ARSU subgroups.^89^

## Results

### Participant Flow

**Figure 1.**
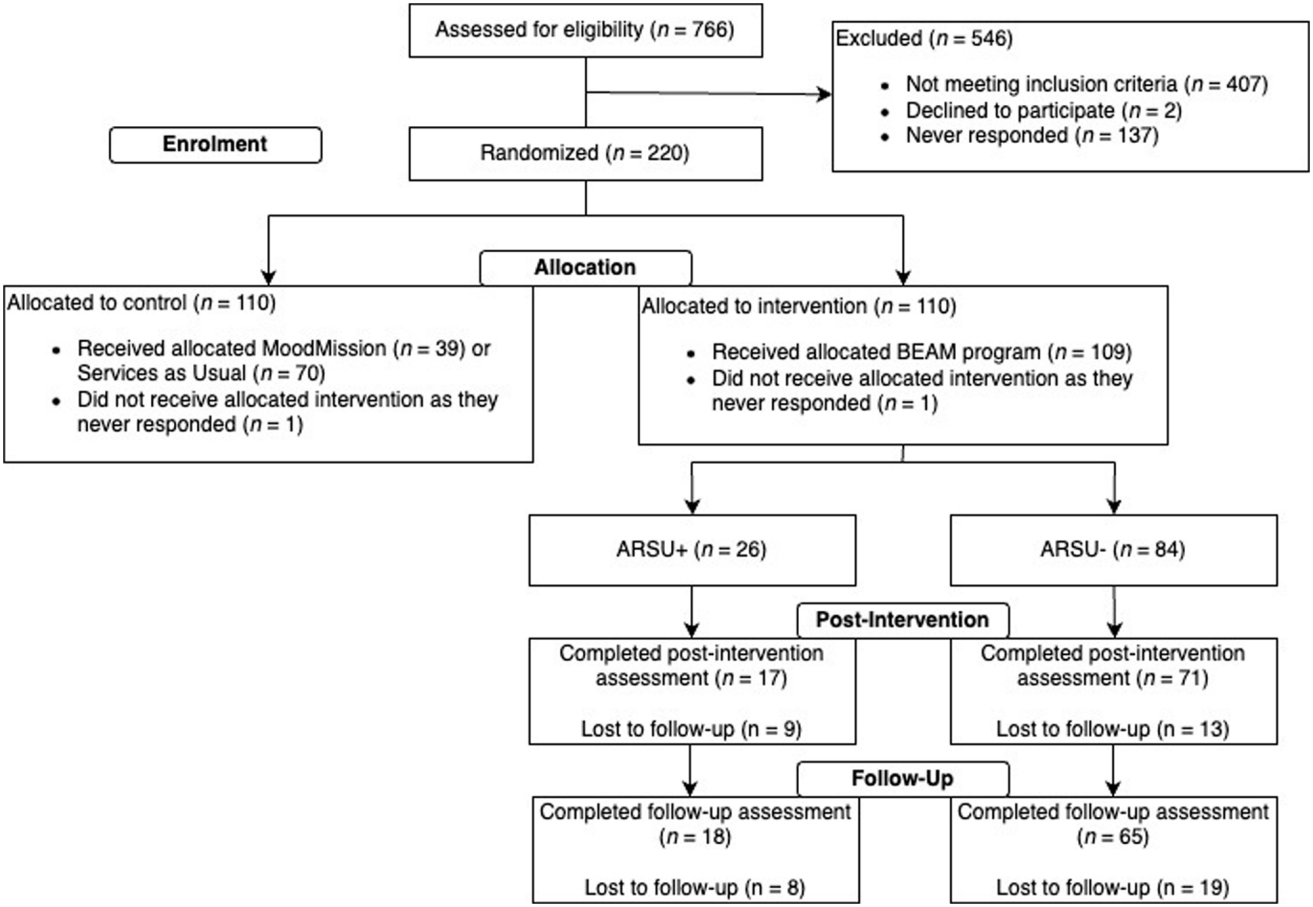
Consolidated Standards of Reporting Trials (CONSORT) flow diagram depicting participation.

Participant enrollment, allocation, and retention are shown in the CONSORT flow diagram (Figure 1). A total of 235 and 531 mothers completed the enrollment screener for the infant and toddler trials, respectively. Of these, 80 (infant) and 140 (toddler) were randomized. Of the 110 mothers in BEAM (infant trial: n = 40; toddler trial: n = 70), there were 26 mothers with ARSU+ and 84 with ARSU−. Attrition did not differ by ARSU subgroup at T2 or T3 (*P*’s > .05).

### Missing Data

Forty-eight participants (43.6%) had at least some missing data, with 15.3% of cells missing overall. The percentage of missing primary outcome data was as follows: anxiety (14.8%), depression (14.8%), sleep (15.2%), anger (15.2%), parenting stress (14.8%), and mental health composite (14.8%). Comparisons of T1 sociodemographic characteristics between participants with and without missing data on primary outcomes revealed no significant differences, suggesting that missingness was not dependent on sociodemographic factors. Missing data were handled using full information maximum likelihood estimation.^90,91^ Data were missing completely at random (MCAR), χ^2^(58) = 70.64, *P* = .12.^92^

### Sociodemographic Characteristics

On average, mothers were 31.85 years old (SD = 4.67) and their index child was 19.67 months old (SD = 8.82). Mothers with ARSU+ were less likely to be married/common law, identify as White, or breast/chest feed than the ARSU− group (*P*’s < .05; Table 3).

**Table 3.** Pre-Intervention Demographics by At-Risk Substance Use Group.

Sociodemographic characteristics	Sample(n = 110)	Group		Group comparison (*t* or χ^2^)
		At-risk substance use (n = 26)	No at-risk substance use (n = 84)	
Trial (Infant, %)	36.4%	30.8%	38.1%	χ^2^ = 0.46, *P* = .50
Child sex (%)				χ^2^ = 0.28, *P* = .59
Female	54.5%	50.0%	56.0%	
Male	45.5%	50.0%	44.0%	
Child age (months) (M, SD)	19.67 (8.82)	21.29 (9.14)	19.20 (8.73)	*t*(36.2) = −1.00, *P* = .33
Mother age (years) (M, SD)	31.85 (4.67)	31.00 (4.80)	32.12 (4.62)	*t*(40.4) = 1.05, *P* = .30
**Married/Common Law (%)**	**77.3**%	**53.8**%	**84.5**%	**χ**^2^ = **10.64, *P* = .001**
Urbanicity (%)	47.2%	45.8%	47.6%	χ^2^ = 0.24, *P* = .88
**White (%)**	**66.1**%	**42.3**%	**73.5**%	**χ** ^2^ **= 8.59, *P* = .003**
Employed (%)	60.6%	48.0%	64.3%	x^2^ =2.14, *P* = .14
Household income > 90K Canadian (%)	43.7%	26.1%	48.8%	χ^2^ = 3.73, *P* = .05
Highest education (%)				χ^2^ = 5.24, *P* = .39
Some high school	5.5%	11.5%	3.6%	
High school	17.3%	23.1%	15.5%	
College/Technical school	30.9%	34.6%	29.8%	
Bachelor’s degree	31.8%	23.1%	34.5%	
Graduate or Professional degree	14.5%	7.7%	16.7%	
No. adults in home (M, SD)	2.03 (0.77)	1.96 (1.00)	2.05 (0.69)	*t*(32.8) = 0.41, *P* = .69
No. children in home (M, SD)	1.91 (1.04)	1.73 (0.78)	1.96 (1.10)	*t*(58.9) = 1.20, *P* = .32
**Breast/Chest feeding (%)**	**45.8**%	**16.7**%	**53.8**%	**χ**^2^ = **7.85, *P* = .005**
Accessed Mental Health or Parenting Resources since Treatment Ended – Follow-Up (%)	36.9%	42.9%	35.7%	χ^2^ = 0.26, *P* = .61
Symptom Severity (Pre) – Percent Possible (M, SD)	0.62 (0.16)	0.66 (0.19)	0.61 (0.15)	*t*(36.2) = −1.23, *P* = .23

*Note.* Significant results are bolded.

### Substance Use Characteristics

Eight (30.8%) mothers in the ARSU+ group met both AUDIT and CUDIT-R cutoffs. At T1, 85 mothers (77.27%) reported alcohol use and 44 (40.00%) used cannabis in the past 6 months. Among those using cannabis, an average of 2.89 (SD = 5.40) standard cannabis joints were used in the past month. Mothers with ARSU+ were more likely to use cannabis at T1 and T2 than the ARSU− group (*P*’s < 0.001). Alcohol use did not differ between ARSU subgroups at any timepoint. In the infant trial, 15.00% of mothers used tobacco products, and none used illicit substances. In the toddler trial, 32.43% used tobacco (M = 8.32 days, SD = 13.12) and 2 used illicit substances in the past month (M = 0.32 days, SD = 1.16).

### Program Engagement

Mothers with ARSU+ attended fewer weekly telehealth groups (*t*_(40.1)_ = 3.12, *P* = .003) and accessed the community forum less often (*t*_(61.6)_= 2.05, *P* = .045) compared to the ARSU− group (see Supplemental Materials A). BEAM was easy to use (*M* = 2.04, SD = 1.41) and had high satisfaction on the MAUQ (*M* = 2.20, SD = 1.38; cutoff score ≤3).

### Implementation Metrics

Implementation benchmarks were met for treatment adherence (>50% of program weeks completed) and safety (no adverse events reported; Table 1) in both ARSU subgroups. The benchmark for perceived usefulness of the BEAM App was met for the ARSU+ subgroup but not the ARSU− group. Neither group met the benchmark for retention (≥80% with follow-up data). Post hoc analyses revealed 31.3% of mothers reported difficulties accessing BEAM psychoeducational videos. Among those, 60% chose not to watch these videos via YouTube when uploaded in response to technical difficulties.

### Symptom Trajectories

There was a positive correlation between T0 symptom severity and anxiety, depression, anger, and sleep disturbance scores at T1 (*P*’s < .001; Table 4) and greater T0 symptom severity was associated with reductions in depression change scores from T1-T2 and T1-T3 (*r* = −.24 and −.23; *P*’s < .05). Results motivated the moderation by T0 symptom severity analysis.^61^

**Table 4.** Correlations Between Sociodemographics and Change in Mental Health Outcomes.

Outcome measure	Married/common law	White	Symptom severity (at eligibility/T0)	Breast/chest feeding
Anxiety (T1)^a^	.066	.098	.494***	−.046
Anxiety (T2-T1)^a^	.191	−.071	−.087	−.073
Anxiety (T3-T1)^a^	.106	−.050	−.128	.017
Depression (T1)^b^	.197*	−.062	.643***	.034
Depression (T2-T1)^b^	.139	−.025	−.238*	−.137
Depression (T3-T1)^b^	.056	−.145	−.226*	−.014
Anger (T1)^c^	−.021	.081	.412***	−.009
Anger (T2-T1)^c^	−.083	.167	−.170	−.101
Anger (T3-T1)^c^	.035	.036	−.030	.051
Sleep (T1)^d^	.073	−.014	.316***	.136
Sleep (T2-T1)^d^	.085	−.030	−.016	−.156
Sleep (T3-T1)^d^	.011	−.074	−.174	−.105
Parenting stress (T1)^e^	.089	−.083	.160	.000
Parenting stress (T2-T1)^e^	−.017	−.034	−.081	−.042
Parenting stress (T3-T1)^e^	−.046	.050	−.097	.070

*Note.* T1, pre-intervention; T2, post-intervention; T3, follow-up; Measured using the ^a^Generalized Anxiety Disorder – 7 Item,^64 b^Patient Health Questionnaire – 9 Item,^63 c^PROMIS-Anger,^75 d^PROMIS-Sleep Disturbance,^76 e^Parenting Stress Index – Short Form.^74^

* *P* < .05. ***P < .001.

#### At-Risk Substance Use Subgroup Characteristics

Table 5 presents the means and standard deviations of each measure by ARSU subgroup over time. The ARSU+ group reported higher T1 hazardous alcohol and cannabis use (AUDIT, CUDIT-R) and T2 (CUDIT-R), consistent with the subgroup definition. No other differences were observed.

**Table 5.** Mental Health and Parenting Stress Outcomes by At-Risk Substance Use Group Across Data Collection Timepoints.

Variable	Overall			At-risk substance use			No at-risk substance use			Clinical
	M (SD)	Min.	Max.	M (SD)	Min.	Max.	M (SD)	Min.	Max.	Cut-offs
*Anxiety* ^a^										⩾10
Pre-intervention	13.75 (5.19)	2	21	14.19 (5.52)	3	21	13.61 (5.11)	2	21	
Post-intervention	8.90 (5.29)	0	21	9.94 (5.67)	2	21	8.65 (5.20)	0	21	
Follow-up	7.63 (5.45)	0	21	8.17 (5.85)	0	21	7.48 (5.37)	0	21	
*Depression* ^b^										⩾10
Pre-intervention	13.78 (5.01)	3	25	14.58 (5.67)	3	25	13.54 (4.80)	4	25	
Post-intervention	9.52 (5.35)	1	24	9.82 (5.09)	3	23	9.45 (5.44)	1	24	
Follow-up	7.99 (5.60)	1	24	8.94 (6.18)	1	24	7.72 (5.45)	1	24	
*Anger* ^c^										⩾16
Pre-intervention	17.45 (2.93)	9	25	17.85 (3.02)	9	23	17.33 (2.91)	9	25	
Post-intervention	15.80 (3.68)	9	25	15.00 (2.92)	10	19	16.00 (3.83)	9	25	
Follow-up	14.72 (4.09)	7	25	14.33 (4.69)	7	25	14.83 (3.94)	8	25	
*Sleep disturbance* ^d^										⩾30
Pre-intervention	28.95 (6.57)	15	40	29.27 (6.48)	17	39	28.84 (6.63)	15	40	
Post-intervention	25.92 (7.03)	8	40	26.50 (8.16)	12	38	25.79 (6.80)	8	40	
Follow-up	23.13 (7.21)	8	40	21.89 (7.45)	8	40	23.48 (7.16)	9	40	
*Parenting Stress Index – Parent-Child Dysfunctional Interaction Subscale* ^e^										-
Pre-intervention	20.02 (5.95)	12	40	19.65 (4.76)	13	32	20.13 (6.29)	12	40	
Post-intervention	19.00 (5.83)	12	36	18.24 (6.03)	13	31	19.18 (5.81)	12	36	
Follow-up	18.86 (6.25)	12	42	20.11 (6.88)	12	42	18.51 (6.08)	12	42	
*Mental health composite*										-
Pre-intervention	0.65 (0.13)	0	1	0.67 (0.15)	0	1	0.64 (0.12)	0	1	
Post-intervention	0.52 (0.16)	0	1	0.54 (0.17)	0	1	0.51 (0.16)	0	1	
Follow-up	0.46 (0.17)	0	1	0.47 (0.19)	0	1	0.46 (0.17)	0	1	
*Alcohol use* (AUDIT)^f^										⩾8
Pre-intervention	3.00 (3.12)	0	17	**5.58 (4.83)**	0	17	**2.20 (1.73)**	0	6	
Post-intervention	2.62 (2.87)	0	15	4.50 (4.60)	0	15	2.20 (2.15)	0	10	
Follow-up	2.70 (3.47)	0	22	4.11 (4.06)	0	16	2.31 (3.22)	0	22	
*Cannabis use* (CUDIT-R)^g^										⩾8
Pre-intervention	2.30 (4.72)	0	23	**8.80 (7.30)**	0	23	**0.72 (1.53)**	0	7	
Post-intervention	2.07 (4.30)	0	22	**6.43 (7.47)**	0	22	**1.20 (2.67)**	0	12	
Follow-up	1.90 (3.61)	0	19	4.00 (6.05)	0	19	1.32 (2.33)	0	10	

*Note.* Measured using the ^a^Generalized Anxiety Disorder – 7 Item,^64^ ^b^Patient Health Questionnaire – 9 Item,^63 c^Patient-Reported Outcomes Measure Information System – Anger Subscale,^75 d^Patient-Reported Outcomes Measure Information System – Sleep Disturbance Subscale,^76 e^Parenting Stress Index – Short Form,^74 f^Alcohol Use Disorder Identification Test,^1^ and ^g^Cannabis Use Disorder Identification Test – Revised^2^; Significant differences between groups are in bold.

#### Main Effects of Time and ARSU Group on Symptom Outcomes

Results were consistent in direction when married/common law status, identifying as White, and breast/chest feeding were included as covariates, and these variables were not associated with symptom change (Table 4). In accordance with the principle of parsimony,^93^ results are presented from models excluding these covariates. Full covariate-adjusted models are provided in Supplemental Materials B. Significant improvements were observed over time across all outcomes: anxiety (*b* = −2.91, SE = 0.37, *P* < .001), depression (*b* = −2.77, SE = 0.36, *P* < .001), anger (*b* = −1.18, SE = 0.25, *P* < .001), sleep disturbance (*b* = −2.60, SE = 0.42, *P* < .001), parenting stress (*b* = −0.88, SE = 0.37, *P* = .018), and the mental health composite (*b* = −0.09, SE = 0.01, *P* < .001; Table 6). Intercepts for depression, anxiety, sleep disturbance, parenting stress, and the mental health composite at T1 varied between participants (*P*’s < .05), whereas anger did not. Variability in symptom change over time was observed for anger and the mental health composite (*P*’s < .01). Random intercept variance indicated heterogeneity in symptom levels at T1 across participants.

**Table 6.** BEAM At-Risk Substance Use Group Treatment Response.

Model effects	Anxiety estimate (SE)	Depression estimate (SE)	Anger estimate (SE)	Sleep disturbance estimate (SE)	Parenting stress estimate (SE)	Mental health composite estimate (SE)
*Random effects*						
Intercept	9.34 (2.31)***	10.87 (2.88)***	1.07 (1.16)	19.84 (4.51)***	12.55 (4.19)**	0.01 (0.00)**
Time	0.60 (0.56)	1.03 (0.73)	1.10 (0.33)**	1.59 (0.95)	0.97 (1.16)	0.00 (0.00)**
*Fixed effects*						
Intercept	15.96 (0.78)***	15.95 (0.73)***	18.47 (0.45)***	31.28 (0.91)***	20.92 (0.88)***	0.72 (0.02)***
Time	−2.91 (0.37)***	−2.77 (0.36)***	−1.18 (0.25)***	−2.60 (0.42)***	−0.88 (0.37)*	−0.09 (0.01)***
ARSU	0.83 (1.72)	0.62 (1.68)	0.72 (0.90)	1.15 (1.65)	−1.73 (1.51)	0.03 (0.04)
*Interaction effects*						
ARSU on time	−0.08 (0.78)	0.23 (0.72)	−0.55 (0.51)	−0.61 (0.69)	0.97 (0.85)	−0.01 (0.02)
Glass’ Δ for ARSU on time^86^	.02	.06	.21	.13	.22	.09
Within-group residual variance	16.50 (1.98)***	13.07 (1.68)***	6.49 (0.75)***	20.57 (2.75)***	18.47 (2.94)***	0.01 (0.00)***

Abbreviation: SE, standard error.

*Note.* Significant results are bolded; ARSU = at-risk substance use.

* *P* < .05. ** P < .01. ***P < .001.

#### Moderation of T0 Symptom Severity

Given the observed associations between T0 symptom severity and T1 mental health outcomes (Table 4), T0 symptom severity was examined as a moderator of symptom change. When added to the model as a covariate and via a time*T0 symptom severity interaction, T0 symptom severity was positively associated with intercept-level anxiety (*b* = 17.87, SE = 4.98, *P* < .001), depression (*b* = 22.90, SE = 3.87, *P* < .001), sleep disturbances (*b* = 17.81, SE = 5.46, *P* = .001), parenting stress (*b* = 12.09, SE = 6.01, *P* = .044), and the mental health composite (*b* = 0.58, SE = 0.11, *P* < .001), but not anger at T1 (*b* = 5.08, SE = 3.11, *P* > .05; Table 7). Symptoms improved over time across ARSU subgroups (anxiety: *b* = −2.95, SE = 0.38, *P* < .001; depression: *b* = −2.81, SE = 0.37, *P* < .001; anger: *b* = −1.15, SE = 0.25, *P* < .001; sleep disturbances: *b* = −2.67, SE = 0.44, *P* < .001; parenting stress: *b* = −0.86, SE = 0.39, *P* = .028; mental health composite: *b* = −0.09, SE = 0.01, *P* < .001). After accounting for T0 symptom severity, there were no ARSU subgroup differences for any outcome. A significant ARSU*T0 symptom severity interaction emerged for parenting stress (*b* = −16.77, SE = 8.29, *P* = .043), indicating a difference in the T1 intercept of parenting stress at average T0 symptom severity between ARSU subgroups. T0 symptom severity did not moderate symptom change (time) and no T0 symptom severity*ARSU group*time interaction was found (*P*’s > .05).

**Table 7.** At-Risk Substance Use Group Treatment Response, Moderated by Enrollment (T0) Symptom Severity.

Model effects	Anxiety estimate (SE)	Depression estimate (SE)	Anger estimate (SE)	Sleep disturbance estimate (SE)	Parenting stress estimate (SE)	Mental health composite estimate (SE)
*Random effects*						
Intercept	3.98 (2.31)	3.20 (2.15)	0.17 (1.01)	15.60 (4.28)***	10.16 (3.74)**	0.00 (0.00)
Time	0.92 (0.57)	1.64 (0.69)*	1.11 (0.35)**	1.91 (0.93)*	0.98 (1.11)	0.00 (0.00)***
*Fixed effects*						
Intercept	16.21 (0.71)***	16.25 (0.63)***	18.52 (0.45)***	31.55 (0.86)***	21.05 (0.91)***	0.73 (0.02)***
Time	−2.95 (0.38)***	−2.81 (0.37)***	−1.15 (0.25)***	−2.67 (0.44)***	−0.86 (0.39)*	−0.09 (0.01)***
Symptom severity^a^	17.87 (4.98)***	22.90 (3.87)***	5.08 (3.11)	17.81 (5.46)**	12.09 (6.01)*	0.58 (0.11)***
ARSU	−0.25 (1.48)	−0.73 (1.40)	0.25 (0.82)	0.07 (1.52)	−1.59 (1.46)	−0.00 (0.03)
*Interaction effects*						
Severity	−0.50 (9.24)	1.98 (6.35)	5.05 (5.29)	−2.77 (7.38)	−16.77 (8.29)*	0.05 (0.21)
ARSU on time	0.06 (0.74)	0.51 (0.66)	−0.49 (0.50)	−0.43 (0.68)	0.95 (0.85)	−0.00 (0.02)
Glass’ Δ for ARSU on time^86^	.02	.15	.19	.10	.22	.03
Symptom severity on time	−2.51 (0.74)	−3.48 (2.29)	0.54 (1.56)	−4.32 (2.81)	−0.95 (2.15)	−0.08 (0.07)
Severity on time	0.54 (3.86)	−4.11 (2.85)	−2.91 (2.33)	3.84 (4.20)	−0.32 (3.58)	−0.06 (0.09)
Within-group residual variance	15.91 (1.91)***	11.82 (1.55)***	6.40 (0.73)***	19.94 (2.62)***	18.43 (2.90)***	0.01 (0.00)***

Abbreviation: ARSU, at-risk substance use; SE, standard error.

*Note.* Significant results are bolded.

a Collected at enrollment (Time 0).

* *P* < .05. **P < .01. ***P < .001.

## Discussion

This was the first study to examine implementation metrics and mental health and parenting stress symptom trajectories within the BEAM arm for mothers with ARSU+ and ARSU−. Findings indicated that several benchmarks were met in the ARSU+ subgroup, including treatment adherence, safety, and perceived usefulness, although the retention benchmark was not met. Across ARSU subgroups, mental health symptoms and parenting stress decreased over time. Exploratory analyses suggested that the relationship between T0 symptom severity and T1 parenting stress differed by ARSU subgroups, but parenting stress improved over time in both groups with no evidence of different trajectories. Findings extend our understanding of eHealth mental health and parenting programs in mothers with ARSU+, a group underrepresented in research. Building on insights from this and prior BEAM trials, BEAM 2.0 incorporates updated content, enhanced delivery methods, and improved platform infrastructure to address previously identified limitations.^94^ BEAM 2.0 is currently being evaluated in a large-scale trial.^94^ Future evaluations that intentionally recruit samples of mothers with ARSU+ should examine whether BEAM refinements improve implementation metrics.

### Implementation Metrics

Some findings supported the implementation of BEAM among both ARSU subgroups, suggesting previous trial results may extend to mothers with ARSU+.^33,35^ Both ARSU subgroups met benchmarks for treatment adherence and safety, suggesting feasibility on these indicators. Findings were generally consistent with those reported for parental mental health treatments. For example, MacKinnon et al’s^30^ meta-analysis found high treatment adherence (defined as >50% session attendance or module completion) in most studies. Although there is limited research on eHealth mental health and parenting skill programs for mothers with ARSU+, the present findings broadly align with outcomes observed in a review of in-person substance use treatments for mothers of young children.^18^

Perceived usefulness scores on the MAUQ showed different patterns by ARSU subgroup: mothers with ARSU+ met the benchmark, whereas those with ARSU− narrowly exceeded the benchmark.^69^ Although these subgroup differences were descriptive, this pattern may reflect differences in treatment expectations, access to alternative supports, or perceived unmet need between subgroups. For example, BEAM’s low-threshold, flexible, and anonymous format may be particularly valued by mothers with ARSU+, a group that is often underrepresented in research.^10^ Technical difficulties encountered during these BEAM trials may also have influenced overall perceived usefulness ratings.^95^ For example, 31.3% of participants reported difficulty accessing BEAM psychoeducational videos, and 60% did not watch the videos via YouTube. Across both trials, BEAM was delivered as a minimum viable product, a simplified version of a product with basic features to allow early user testing and refinement.^49,50,96,97^ Future qualitative research with mothers and the PAB will be important to identify opportunities to improve BEAM’s functionality and perceived usefulness through targeted modifications.

The retention rate benchmark was not met in either ARSU subgroup (benchmark: ≥80%). While some studies involving high-barrier and underserved populations report retention rates >60% are often considered acceptable,^11,22,98^ retention (ARSU+: 69.2% vs ARSU−: 77.4%) remained below the ideal 80% threshold. Although we did not formally collect data on whether participating mothers were classified as high-barrier, literature suggests mothers with ARSU+ often face systematic barriers and are considered underserved in clinical contexts.^11,22^ In-person substance use treatments for mothers with substance use disorders often report higher retention rates (80.6%-91.7%),^18^ whereas eHealth treatments for parental mental health typically achieve retention rates that are comparable to those observed here.^30^ Taken together, findings highlight retention as a key target for improvement in future trials among mothers with ARSU+, and prior eHealth studies suggest using strategies such as increasing compensation over time,^99-101^ collecting secondary contact details to reduce attrition,^102^ and using technology-enhanced reminders.^103^

### Symptom Trajectories for Mothers With ARSU+

In this exploratory subgroup analysis, there was no evidence of differential mental health or parenting stress symptom change between ARSU subgroups. These findings provide preliminary evidence that symptom trajectories observed in prior BEAM trials^33,35,49,50^ may also be observed among mothers with ARSU+. However, because a BEAM versus TAU comparison could not be conducted in the ARSU+ subgroup due to limited power, causal inferences about BEAM’s effectiveness in this group cannot be drawn. Instead, results provide early insight into symptom trajectories that align with meta-analyses demonstrating small-to-medium effects of eHealth treatments on mental health symptoms in pregnant and postpartum people, including depression (*g* = 0.29 and 0.37), anxiety (*g* = 0.26 and 0.53), and parenting stress (*g* = 0.33).^30,31^ Other meta-analyses have found substance use reductions for pregnant people receiving eHealth treatments (OR = 1.33).^104^ However, substance use outcomes could not be assessed here because these data were not collected as part of the parent trials. Future BEAM trials should include direct substance use measures and continue leveraging partnerships with the PAB to ensure mothers with lived experience of ARSU+ and trusted community partners are meaningfully involved.

### Limitations and Considerations for Interpretation

The impact of BEAM on substance use quantity and frequency could not be examined. Future studies should recruit larger samples of mothers with ARSU+ and use consistent, validated measures to assess BEAM’s impact on substance use (eg, Timeline Follow Back).^105,106^ Additionally, the ARSU+ subgroup sample size precluded examination of whether implementation outcomes varied by T1 AUDIT or CUDIT-R scores. Future trials with larger ARSU+ samples should evaluate whether AUDIT and/or CUDIT-R scores predict differential engagement with BEAM and attainment of implementation metric benchmarks. Second, many participants reported an annual household income >$90K CAD (43.7%) and having at least a high school diploma (94.5%), which may limit generalizability to mothers of lower socioeconomic status.^107^ Despite this, participants were generally representative of the Canadian population in terms of household income and marital status.^108,109^ Education levels were slightly higher (94.5% with at least a high school diploma vs 86.3% in Canada), while those identifying as White/Caucasian was slightly lower (66.1% vs 69.8% in Canada).^110,111^ Third, technical difficulties affected psychoeducational videos access. As such, some mothers may not have received adequate exposure to BEAM’s therapeutic content. To address this, BEAM 2.0 was developed to improve cross-platform functionality and incorporate feedback from prior iterations,^35,61^ patient-partners, and community partners.^94^ Fourth, trials were conducted during the COVID-19 pandemic. Observed symptom reductions may therefore reflect broader changes in mental health symptoms and pandemic-related stressors over time.^112^ Further RCTs are needed to determine the feasibility of BEAM in post-pandemic contexts. Finally, eligibility criteria in the infant trial included MINI administration and potential exclusion due to psychotic symptoms, post-traumatic stress disorder, or an alcohol/substance use disorder. This eligibility criteria may have limited generalizability to mothers with acute or complex clinical presentations; however, no mothers were excluded for endorsing the same on the MINI.

### Strengths and Opportunities for Future Adaptation

This was the first study to examine the feasibility and symptom trajectories of BEAM among mothers with ARSU+, a population that has historically faced limited access to acceptable, evidence-based mental health and parenting programs.^11,18,19,113,114^ Although we could not compare outcomes among mothers with ARSU+ in BEAM versus TAU, precluding conclusions about effectiveness, this study offers early insight into the feasibility and potential relevance of BEAM for this underserved group. BEAM was offered with no treatment-specific costs to participants (assuming access to the internet and a smartphone) and was designed to overcome common structural barriers such as cost, transportation, and scheduling conflicts.^115^ Importantly, BEAM incorporated lay mental health workers (peer and parent coaches), aligning with global recommendations.^116^

### Future Directions

Although BEAM was not originally designed to target substance use, future iterations could incorporate strategies to better support the needs of mothers with ARSU+, as identified by findings from this study and the broader literature. This sample comprised community-recruited mothers with ARSU+ who were not engaged in specialized substance use disorder treatment, programs that often serve individuals with more severe and complex substance use profiles beyond alcohol or cannabis use alone, and higher clinical acuity. BEAM may offer a promising avenue to address key gaps in integrated mental health and parenting supports for mothers with ARSU+ in community and perinatal mental health settings, particularly as a low-intensity option that can be aligned with stepped-care pathways, if effectiveness is established in future trials. Any future use of BEAM as an adjunct within perinatal substance use treatment programs would require dedicated evaluation in those contexts, and our current findings should not be interpreted as directly generalizable to high-intensity substance use treatment settings (Table 8).

**Table 8.** Future Directions and Recommendations.

Future direction	Finding	Recommendation
Lactation and Harm-Reduction Psychoeducation	A meaningful subset of mothers with ARSU+ reported breast/chest feeding (16.7%).	Include brief, harm-reduction psychoeducation on alcohol and cannabis use during lactation (eg, how to reduce infant exposure, timing considerations, decision support), co-designed with mothers with lived experience of ARSU+ to ensure content is empathetic and non-stigmatizing.
Engagement and Retention Optimization	Mothers with ARSU+ attended fewer weekly telehealth groups and used the community forum less often than the ARSU− subgroup. In addition, the pre-specified retention benchmarks were also not met for either subgroup.	Implement low-burden, non-stigmatizing engagement supports to reduce attrition and increase participation which are co-designed with mothers with lived experience of ARSU+. Examples include increasing compensation over time,^99-101^ collecting secondary contact details to reduce attrition,^102^ and using technology-enhanced reminders.^103^
Culturally Grounded Practices	Sociodemographic differences were observed between ARSU subgroups, with fewer participants who identify as White in the ARSU+ (42.3%) vs ARSU− subgroup (73.5%).	Future adaptations should include culturally grounded approaches to support acceptability and perceived safety across diverse groups. While cultural fit was not directly assessed in this study, co-developing content with community partners, Indigenous Knowledge Keepers, and mothers with lived experience of ARSU+ can help ensure BEAM is culturally relevant and responsive, factors that may strengthen engagement and retention.
Measurement of Substance Use Outcomes	Substance use quantity, frequency, and change over time were not measured as outcomes, limiting the ability to characterize substance use trajectories.	Future trials should include standardized, validated measures of alcohol and cannabis use (eg, the Timeline Follow Back^105,106^) to characterize substance use patterns over time.
Qualitative Research and Parent Advisory Boards	The quantitative data presented here provide limited insight into acceptability, relevance, and barriers to engagement that may affect mothers with ARSU+.	Qualitative research should be conducted to ensure that future iterations of BEAM are grounded in the lived experiences of mothers with ARSU+. Interviews and focus groups could help glean valuable insight into priorities, preferences, and perceived barriers to treatment accessibility. These findings could also inform adaptations to treatment content, ensuring it is well accepted and relevant to this population. Additionally, establishing a PAB composed of mothers with lived experience of ARSU+ will support co-designed treatment content, provide ongoing feedback throughout treatment adaptations, and help ensure cultural safety and relevance.

## Conclusion

This study provides preliminary evidence on the feasibility and promise of delivering BEAM to mothers with ARSU+. Reductions in depression, anxiety, anger, sleep disturbances, a mental health composite, and parenting stress were observed over time. Improvements in mental health and parenting stress were similar between ARSU subgroups, even after controlling for T0 symptom severity. While findings suggest BEAM may be feasible and beneficial for mothers with ARSU+, it is important to note that a comparison between BEAM and TAU could not be conducted due to insufficient power. Therefore, causal inferences about BEAM’s effectiveness cannot be made. To improve feasibility for mothers with ARSU+, future adaptations should focus on improving retention rate. Overall, BEAM offers a scalable, evidence-based treatment that holds promise, pending further evaluation, to address unmet mental health and parenting needs among mothers with ARSU+ in Canada.

## Supplemental Material

sj-docx-1-sat-10.1177_29768357261438586 – Supplemental material for Bridging the Gap: A Secondary Data Analysis of Implementation Outcomes and Symptom Trajectories of an eHealth Mental Health and Parenting Treatment Among Mothers With and Without At-Risk Substance Use Supplemental material, sj-docx-1-sat-10.1177_29768357261438586 for Bridging the Gap: A Secondary Data Analysis of Implementation Outcomes and Symptom Trajectories of an eHealth Mental Health and Parenting Treatment Among Mothers With and Without At-Risk Substance Use by Kayla M. Joyce, Robert J. W. McHardy, Lauren E. Kelly, Kristin Reynolds, Natalie Mota, Lianne M. Tomfohr-Madsen and Leslie E. Roos in Substance Abuse: Research and Treatment
