# Supplementary material for: Bridging the Gap: A Secondary Data Analysis of Implementation Outcomes and Symptom Trajectories of an eHealth Mental Health and Parenting Treatment Among Mothers With and Without At-Risk Substance Use
Source: Subst Use. 2026 Apr 9;20:29768357261438586. doi: 10.1177/29768357261438586 (PMC13069170; doi:10.1177/29768357261438586)
Supplement: sj-docx-1-sat-10.1177_29768357261438586 – Supplemental material for Bridging the Gap: A Secondary Data Analysis of Implementation Outcomes and Symptom Trajectories of an eHealth Mental Health and Parenting Treatment Among Mothers With and Without At-Risk Substance Use [file sj-docx-1-sat-10.1177_29768357261438586.docx]

**Supplemental Materials A**

***Program Engagement***

Table A.1 presents BEAM program engagement metrics. On average, all BEAM participants completed 7.90 weekly symptom surveys, attended 5.95 weekly telehealth group sessions, viewed 3.18 mental health videos (at least halfway; out of 10), and viewed 2.37 parenting videos (at least halfway; out of 10). Mothers with ARSU+ attended fewer weekly telehealth groups (*t*_(40.1)_ = 3.12, *p*=.003) and accessed the community forum less often (*t*_(61.6)_ = 2.05, *p*=.045), compared to mothers in the ARSU- group.

| **Table A.1.** Means and standard deviations of the BEAM program engagement metrics | | | |
| --- | --- | --- | --- |
| **Engagement Metric** | **BEAM** | | |
|  | **Total Sample**  ***M* (*SD*)** | **ARSU+**  ***M* (*SD*)** | **ARSU-**  ***M* (*SD*)** |
| Number of Weekly Surveys Completed | 7.90 (3.30) | 6.50 (3.63) | 8.47 (3.01) |
| **Total Community Forum Views** | 70.08 (110.25) | **38.61 (79.63)** | **79.94 (116.89)** |
| Time on Mental Health Videos (in minutes) | 37.93 (51.38) | 30.04 (46.07) | 40.43 (52.97) |
| Total Mental Health Videos Watched ≥ 50% | 3.18 (3.49) | 2.25 (3.17) | 3.55 (3.55) |
| Time on Parenting Videos (in minutes) | 27.22 (39.97) | 17.20 (30.79) | 30.36 (42.12) |
| Total Parenting Videos Watched ≥ 50% | 2.37 (3.06) | 1.25 (2.49) | 2.65 (3.15) |
| **Number of BEAM Telehealth Group Sessions Attended** | 5.95 (3.55) | **4.08 (3.58)** | **6.56 (3.35)** |
| *Note:* Significant differences between groups are in bold. | | | |

**Supplemental Materials B

*Full Model Results When Covariates are Included***

| **Table B.1.** At-risk substance use group treatment response | | | | | | |
| --- | --- | --- | --- | --- | --- | --- |
|  | **Anxiety Estimate (*SE*)** | **Depression Estimate (*SE*)** | **Anger Estimate (*SE*)** | **Sleep Disturbance Estimate (*SE*)** | **Parenting Stress Estimate (*SE*)** | **MH Comp. Estimate (*SE*)** |
| *Random effects* |  |  |  |  |  |  |
| Intercept | **8.60 (2.32)^***^** | **9.86 (2.84)^**^** | 0.93 (1.19) | **19.75 (4.57)^***^** | **12.21 (4.08)^**^** | **0.01 (0.00)^**^** |
| Time | 0.52 (0.53) | 0.99 (0.74) | **1.09 (0.32)^**^** | 1.43 (0.99) | 0.96 (1.14) | **0.00 (0.00)^**^** |
| *Fixed Effects* |  |  |  |  |  |  |
| Intercept | **15.78 (1.81)^***^** | **17.78 (1.99)^***^** | **17.87 (0.97)^***^** | **29.60 (2.11)^***^** | **24.13 (2.35)^***^** | **0.72 (0.05)^***^** |
| Time | **-1.98 (0.85)^*^** | **-2.21 (0.86)^*^** | **-1.09 (0.55)^*^** | -0.76 (0.92) | **-1.91 (0.98)^†^** | **-0.06 (0.02)^*^** |
| Married/Common Law | -0.88 (1.50) | -2.67 (1.78) | 0.60 (0.85) | -0.85 (2.10) | -1.01 (2.15) | -0.04 (0.04) |
| Breast/Chest Feeding | -0.62 (1.55) | -0.43 (1.50) | -0.59 (0.84) | 2.34 (1.78) | -1.42 (1.74) | 0.00 (0.04) |
| White | 1.60 (1.44) | 0.81 (1.48) | 0.55 (0.85) | 1.37 (1.68) | -2.13 (1.91) | 0.04 (0.03) |
| ARSU | 0.91 (1.93) | -0.07 (1.55) | 0.93 (0.94) | 2.00 (1.77) | -3.17 (1.90) | 0.04 (0.04) |
| *Interaction Effects* |  |  |  |  |  |  |
| Married/Common Law on Time | -0.91 (0.69) | -0.14 (0.75) | -0.37 (0.56) | -0.65 (0.87) | 0.06 (1.00) | -0.02 (0.02) |
| Breast/Chest Feeding on Time | -0.02 (0.68) | -0.00 (0.66) | 0.11 (0.44) | -0.98 (0.77) | 0.89 (0.72) | -0.01 (0.02) |
| White on Time | -0.10 (0.66) | -0.52 (0.66) | 0.23 (0.49) | -0.88 (0.74) | 0.66 (0.85) | -0.01 (0.02) |
| ARSU on Time | -0.41 (0.94) | 0.04 (0.73) | -0.57 (0.55) | -1.38 (0.74) | 1.51 (0.97) | -0.02 (0.02) |
| Glass’ Δ for ARSU on Time^85^ | .10 | .01 | .22 | .31 | .35 | .20 |
| Within-group Residual Variance | **16.47 (1.98)^***^** | **12.98 (1.67)^***^** | **6.45 (0.75)^***^** | **20.29 (2.63)^***^** | **18.23 (2.88)^***^** | **0.01 (0.00)^***^** |
| *Note*: Employment and breast/chest feeding were added the model; †*p* < .10. ^*^*p* < .05. ^**^ *p* < .01. ^***^*p*< .001. | | | | | | |

| **Table B.2.** At-risk substance use group treatment response, moderated by enrollment (T0) symptom severity | | | | | | |
| --- | --- | --- | --- | --- | --- | --- |
|  | **Anxiety Estimate (*SE*)** | **Depression Estimate (*SE*)** | **Anger Estimate (*SE*)** | **Sleep Disturbance Estimate (*SE*)** | **Parenting Stress Estimate (*SE*)** | **MH Comp. Estimate (*SE*)** |
| *Random effects* |  |  |  |  |  |  |
| Intercept | 3.26 (2.18) | 1.86 (2.01) | 0.00 (0.04) | **15.37 (4.29)^***^** | **9.92 (3.73)^**^** | 0.00 (0.00) |
| Time | 0.82 (0.55) | **1.67 (0.70)** | **1.11 (0.24)^***^** | 1.71 (0.95) | 1.01 (1.09) | **0.00 (0.00)^***^** |
| *Fixed Effects* |  |  |  |  |  |  |
| Intercept | **16.30 (1.60)^***^** | **18.92 (1.52)^***^** | **18.26 (0.93)^***^** | **30.05 (2.06)^***^** | **24.02 (2.32)^***^** | **0.74 (0.04)^***^** |
| Time | **-1.85 (0.83)^*^** | **-2.56 (0.81)^**^** | **-1.16 (0.57)^*^** | -0.61 (0.89) | -1.97 (1.04) | **-0.06 (0.02)^**^** |
| Married/Common Law | -0.62 (1.38) | **-2.78 (1.42)** | 0.46 (0.84) | -0.49 (2.03) | -0.60 (2.14) | -0.04 (0.03) |
| Breast/Chest Feeding | -1.06 (1.40) | -0.94 (1.23) | -0.77 (0.80) | 1.96 (1.69) | -1.55 (1.79) | -0.02 (0.03) |
| White | 1.23 (1.31) | 0.22 (1.28) | 0.40 (0.86) | 0.95 (1.72) | -2.13 (1.88) | -0.03 (0.03) |
| Symptom Severity | **17.74 (4.99)^***^** | **22.06 (3.94)^***^** | 5.36 (3.18) | **17.07 (5.66)^**^** | **12.23 (5.97)^*^** | **0.57 (0.11)^***^** |
| ARSU | -0.34 (1.70) | -1.88 (1.32) | 0.30 (0.88) | 0.87 (1.71) | -3.05 (1.87) | -0.01 (0.03) |
| *Interaction Effects* |  |  |  |  |  |  |
| ARSU * Symptom Severity | -0.73 (9.31) | 4.81 (6.55) | 4.39 (5.53) | -2.95 (7.79) | -14.89 (8.49) | 0.07 (0.21) |
| Married/Common Law on Time | -1.11 (0.68) | 0.05 (0.76) | -0.25 (0.58) | -0.99 (0.83) | 0.14 (1.04) | -0.02 (0.02) |
| Breast/Chest Feeding on Time | 0.03 (0.68) | 0.07 (0.65) | -0.12 (0.43) | -0.92 (0.75) | 0.91 (0.73) | -0.01 (0.02) |
| White on Time | -0.11 (0.67) | -0.38 (0.68) | 0.21 (0.50) | -0.81 (0.77) | 0.64 (0.87) | -0.01 (0.02) |
| ARSU on Time | -0.34 (0.89) | 0.43 (0.72) | -0.48 (0.54) | -1.34 (0.73) | 1.55 (0.98) | -0.01 (0.02) |
| Glass’ Δ for ARSU on Time^85^ | .09 | .12 | .19 | .30 | .36 | .15 |
| Symptom Severity on Time | -2.70 (2.63) | -3.29 (2.33) | 0.40 (1.58) | -4.09 (2.83) | -1.13 (2.10) | -0.08 (0.07) |
| ARSU * Symptom Severity on Time | 1.83 (3.83) | -4.06 (3.03) | -2.71 (2.45) | 5.22 (4.22) | -0.79 (3.77) | -0.04 (0.09) |
| Within-group Residual Variance | **15.89 (1.89)^***^** | **11.75 (1.51)^***^** | **6.37 (0.70)^***^** | **19.76 (2.53)^***^** | **18.14 (2.82)^***^** | **0.01 (0.00)^***^** |
| *Note*: Employment and breast/chest feeding were added the model; †*p* < .10. ^*^*p* < .05. ^**^ *p* < .01. ^***^*p*< .001. | | | | | | |
